# Overexpression of a WRKY Transcription Factor *TaWRKY2* Enhances Drought Stress Tolerance in Transgenic Wheat

**DOI:** 10.3389/fpls.2018.00997

**Published:** 2018-08-07

**Authors:** Huiming Gao, Yafei Wang, Ping Xu, Zhengbin Zhang

**Affiliations:** ^1^Key Laboratory of Agricultural Water Resources, Hebei Laboratory of Agricultural Water-Saving, Center for Agricultural Resources Research, Institute of Genetics and Developmental Biology, Chinese Academy of Sciences, Shijiazhuang, China; ^2^University of Chinese Academy of Sciences, Beijing, China

**Keywords:** *TaWRKY2*, transgenic wheat, transcription factors, yield, drought

## Abstract

Drought is a major environmental stress that severely restricts plant growth and crop productivity. A previous study showed that *TaWRKY2* from wheat (*Triticum aestivum*) plays an important role in drought stress tolerance. In the present study, we isolated the promoter of *TaWRKY2* and identified multiple regulatory *cis*-elements in the promoter region. The activity of the *TaWRKY2* promoter was induced by drought, salt, heat, and abscisic acid (ABA). We also generated *TaWRKY2*-overexpressing transgenic wheat, and found that the transgenic seedlings exhibited significantly enhanced tolerance to drought stress, as evidenced by a higher survival rate and lower water loss rate of detached leaves compared with wild type (WT) plants. In addition, the transgenic lines had higher contents of free proline, soluble sugar, and chlorophyll. During a prolonged period of drought stress before the heading stage, the growth of WT plants was inhibited, whereas the *TaWRKY2*-overexpressing lines progressed to the heading stage. The increased grain yield of the transgenic wheat lines reflected the cumulative effects of longer panicle length, more kernels per spike, and greater aboveground biomass. Our findings show that *TaWRKY2* can enhance drought tolerance and increase grain yield in wheat, thus providing a promising candidate target for improving the drought tolerance of wheat cultivars through genetic engineering.

## Introduction

Wheat (*Triticum aestivum*) is one of the most important staple crops worldwide, and its production in many regions is severely impacted by drought; thus, improving the drought tolerance of wheat through the breeding of cultivars is an important step toward ensuring food security.

Reflecting their sessile nature, plants have developed a fairly complex stress response system to deal with drought via changes at the physiological, morphological, and molecular levels (Todaka et al., 2012; Lata et al., 2015; Tiwari et al., 2017). Transcription factors (TFs) can synchronously regulate the expression of many downstream drought-regulated genes and thus mediate the response to drought in plants (Singh and Laxmi, 2015; Gahlaut et al., 2016). The overexpression of some stress-responsive TFs can enhance abiotic stress tolerance in transgenic plants (Saad et al., 2013). For instance, the *Arabidopsis* homeodomain-leucine zipper TF gene *EDT1/HDG11* not only conferred drought tolerance, but also increased grain yield in transgenic rice (Yu et al., 2013). The *Oryza sativa* TF gene *OsMYB2* was found to be induced by salt and cold, and transgenic rice overexpressing *OsMYB2* showed increased tolerance to various abiotic stresses by regulating the relative expression levels of diverse genes involved in abiotic stress responses (Yang et al., 2012).

With the rapid development of genetic engineering to improve abiotic stress tolerance in plants, TFs may be a practical and an effective class of targets for improving stress tolerance in wheat. Several examples from previous studies using transgenic wheat are described below. *TaERF3*-overexpressing transgenic wheat seedlings exhibited significantly enhanced tolerance to both salt and drought stresses compared with untransformed wheat (Rong et al., 2014). *TaPIE1* overexpression significantly enhanced resistance to freezing stress (Zhu et al., 2014). *TaHsfC2a*-overexpressing transgenic wheat showed improved thermal tolerance (Hu et al., 2017). *GmDREB1*, a soybean TF gene, was also shown to improve salt stress tolerance in transgenic wheat (Jiang Q. et al., 2014).

The WRKY TFs family not only imparts tolerance against abiotic stresses, but also regulates plant growth and development (Jiang et al., 2012; Zhu et al., 2013; Tripathi et al., 2014; Xu et al., 2014). In recent years, many *WRKY* genes have been identified in a variety of plants (Fan et al., 2015; He et al., 2016; Xu et al., 2016; Wei et al., 2017). Recently, most studies of WRKY TFs have based on functional analyses of responses to abiotic stress, such as drought and salt, with functional validation conducted only in the model plants (Wang et al., 2013; Xu et al., 2014; Qin et al., 2015). However, functional validation of the vast majority of WRKYs has yet to be confirmed in non-model plants, particularly wheat, in which WRKY-mediated drought resistance mechanisms have not been studied. We previously reported that *TaWRKY2* was induced in response to drought stress. Additionally, overexpression of *TaWRKY2* conferred tolerance to drought stress in transgenic *Arabidopsis* (Niu et al., 2012). However, to the best of our knowledge, the roles of *TaWRKY2* in the drought stress response of wheat and its functional mechanisms in wheat have not been reported.

To investigate how *TaWRKY2* induces drought stress responses, we cloned the promoter of the *TaWRKY2* gene and identified the regulatory *cis*-elements in the promoter region in the present study. Transgenic wheat generated by conducting *Agrobacterium*-mediated transformation with *TaWRKY2*-overexpressing vectors were used to confirm the function of *TaWRKY2*, and the physiological mechanisms of drought resistance were analyzed. In addition, the effects of *TaWRKY2* overexpression on wheat yield were studied. Taken together, the present findings indicate that *TaWRKY2* contributes to drought tolerance and increases yield.

## Materials and Methods

### Plant Materials

The promoter of *TaWRKY2* (NCBI ID: EU665425.1) was cloned from Xifeng20, a common drought-tolerant wheat variety. Fielder, a spring wheat variety, was used as the recipient for the *TaWRKY2*-overexpressing transformation. T_1_–T_3_ transgenic wheat plants. *Nicotiana benthamiana* were used for β-glucuronidase (GUS) histochemical analysis.

### Cloning of the *TaWRKY2* Promoter and GUS Histochemical Analysis

The *TaWRKY2* promoter was cloned from Xifeng20 using specific primers (P_W2_-F and P_W2_-R). PCR was performed in a 50-μl reaction volume containing 200 ng genomic DNA, 0.4 μM dNTPs, 0.3 μM each primer and 1 U KOD FX (Toyobo, Shanghai, China). The PCR program used was 94°C for 2 min; 40 cycles of 98°C for 10 s, 55°C for 30 s, and 68°C for 2.5 min; final extension at 72°C for 10 min; and storage at 4°C. The cloned sequence was inserted into pCAMBIA1391 to obtain P*_TaWRKY 2_*::GUS for GUS histochemical analysis. The P*_TaWRKY 2_*::GUS construct was introduced into *Agrobacterium tumefaciens* strain GV3101 and transformed into *N. benthamiana* leaves. Two days after being cultured under 14 h light/10 h dark at 25°C, the injected *N. benthamiana* was treated with different abiotic stresses. The GUS assay was performed as previously described (Zhao et al., 2011). The histochemical staining was conducted by incubating the materials in prepared buffer containing 5-bromo-4-chloro-3-indolyl-β-D-glucuronic acid for 12 h at 37°C in the dark followed by clearing with 75% ethanol.

### Overexpression Vector Construction and Transformation

A 1,407-bp fragment corresponding to the *TaWRKY2* coding sequence (CDS) was amplified from the vector pBIN121-*TaWRKY2* that was introduced into *Arabidopsis thaliana* (Columbia) (Niu et al., 2012) with specific primers (Ta2-F and Ta2-R). The CDS was subsequently cloned into the *Spe*I and *Bam*hI sites of pMWB122, a binary expression vector with an ubiquitin promoter. The recombinant vector pMWB122-*TaWRKY2* was transformed into a spring wheat variety, Fielder, using *Agrobacterium*-mediated transformation. The transformation was performed by Xingguo Ye’s group from the Chinese Academy of Agricultural Sciences.

### PCR and Quantitative Reverse Transcription-PCR Analyses

Genomic DNA was isolated from leaves of wheat using the cetyl-trimethyl-ammonium bromide (CTAB) method. A 381-bp fragment was amplified by PCR using specific primers (TaW2-F and TaW2-R). PCR reactions were performed in a 20-μl reaction volume, containing 50 ng wheat genomic DNA, 0.4 μM of each primer, and 1 U Taq polymerase (TransGen Biotech, Beijing, China). The PCR program used was 94°C for 2 min; followed by 32 cycles of 94°C for 30 s, 56°C for 30 s, and 72°C for 40 s; final extension at 72°C for 10 min; and storage at 4°C. The amplified fragments were separated on a 1.0% agarose gel.

Total RNA was extracted from wheat leaves using TRIZOL reagent (TransGEN, Beijing, China). cDNA was synthesized using the TansSript kit (TransGEN, Beijing, China). qRT-PCR was used to analyze transcript levels of *TaWRKY2* (QT-Ta2-F and QT-Ta2-R) and eight drought-related genes following the methods described in Niu et al. (2012). Wheat *Actin* was used as an internal reference to normalize all data (Taactin F and Taactin R). All primers were listed in **Supplementary Table S1**.

### Southern Blot

The transgenic *TaWRKY2* gene copy number was determined by Southern blot. Genomic DNA was extracted from the leaves of 3-week-old transgenic wheat plants by using the CTAB method (Sambrook et al., 1989). Each sample was digested with *Hind*III (TaKaRa, Dalian, China) overnight, and the digested DNA was electrophoresed on 0.8% agarose gels and then transferred onto a nylon Hybond-N^+^ membrane (Roche). The DNA fragments of the ubiquitin promoter were amplified with specific primers (UbiF and UbiR; **Supplementary Table S1**) and were labeled with digoxigenin (DIG) and used as probes to hybridize with the digested DNA on the membrane. The hybridization and detection steps were performed following the instructions for the DIG High Prime DNA Labeling and Detection Starter Kit II (Roche).

### Drought Treatment and Measurement of Drought-Related Indicators

For the drought tolerance assays at the seedling stage, the wheat plants were cultured in plastic containers (depth: 8 cm, diameter: 8 cm) containing mixed soil (1:1 nutritional soil:vermiculite), with nine plants per pot. All plants were grown in a culture room under standard growth conditions (14 h light at 24°C/10 h dark at 18°C). After the full development of the three leaves of each plant, the wheat seedlings were subjected to drought treatment by withholding water for 8–11 days before re-watering. Meanwhile, the soil relative water content (SRWC) and leaf relative water content (LRWC) were calculated as formulas in **Supplementary Table S3**. The water loss rate of detached leaves was calculated as previously described (Niu et al., 2012). Free proline, chlorophyll, soluble sugar, and H_2_O_2_ contents in leaves were measured following the kit introductions (Comin, Suzhou, China). Each data point corresponds to three replicates.

For the analysis of *TaWRKY2* effects on the heading stage, wheat plants were cultured in the plastic containers (depth: 30 cm, diameter: 25 cm) in a greenhouse. Approximately 20–25 days before the heading stage, the following treatments were applied: (1) wild type (WT) and transgenic wheat plants were grown under normal condition until harvest, and (2) the same plants were subjected to 20–25 days of water-withholding (drought treatment), followed by re-watering until harvest. The entire experiment was carried out at room temperature.

Agronomic traits data were collected from the harvested transgenic wheat and WT plants under normal and drought conditions mentioned above for statistical analysis.

## Results

### Analysis of *TaWRKY2* Promoter

**FIGURE 1:**
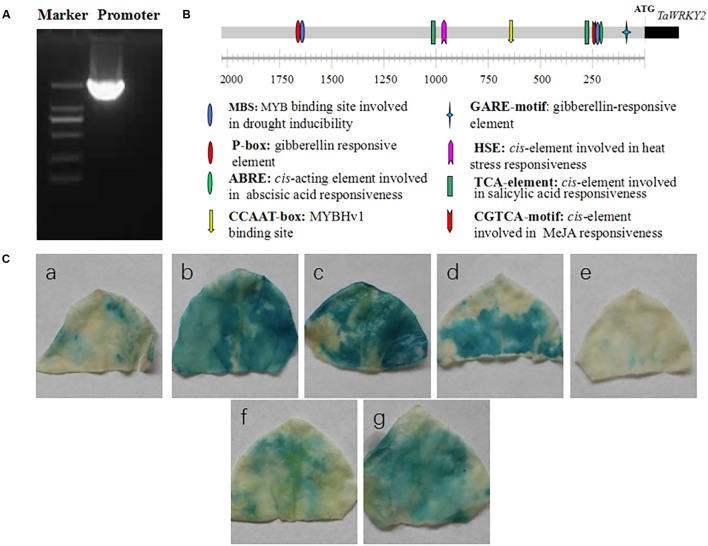
Promoter cloning and analysis of *TaWRKY2.* **(A)** The 2,166-bp promoter upstream of the ATG start codon of *TaWRKY2*. **(B)** Analysis of the *cis*-regulatory promoter elements. **(C)** Responsiveness of the promoter to the following abiotic stresses: **(a)** control, **(b)** drought (20% polyethylene glycol for 24 h), **(c)** salt (300 mM NaCl for 24 h), **(d)** heat (37°C for 12 h), **(e)** cold (4°C for 12 h), **(f)** sprayed with H_2_O for 24 h, and **(g)** sprayed with ABA (200 mM for 24 h).

It has been found that the expression of *TaWRKY2* was induced by drought (Niu et al., 2012). To further understand the mechanism underlying this phenomena, we cloned the 2,166-bp DNA fragment upstream of the ATG start codon of *TaWRKY2* from the wheat variety Xifeng20 (**Figure 1A**). Additionally, we analyzed the promoter region to identify regulatory *cis*-elements using PlantCARE^1^. As shown in **Figure 1B** and **Supplementary Table S2**, numerous *cis*-elements were identified in the *TaWRKY2* promoter region. Consistent with the hypothesized function in stress tolerance, one abscisic acid (ABA)-responsive element (ABRE) *cis*-element involved in ABA response and two MBS *cis*-elements (V-myb avian myeloblastosis viral oncogene homolog (MYB) binding site) involved in drought response were found in the *TaWRKY2* promoter. Unexpectedly, the *TaWRKY2* promoter also contained *cis*-elements involved in plant hormone signaling with functions in plant growth regulation and stress response, including elements associated with gibberellic acid (GA), methyl jasmonate acid (MeJA), and salicylic acid (SA). These findings suggest that *TaWRKY2* plays an important role in plant responses to drought and that it may also modulate plant development through hormone regulation under stress conditions. GUS histochemical analysis showed that the activity of the *TaWRKY2* promoter was up-regulated by drought, salt, heat, and ABA (**Figure 1C**).

### Molecular Characterization of Transgenic Wheat Plants

**FIGURE 2:**
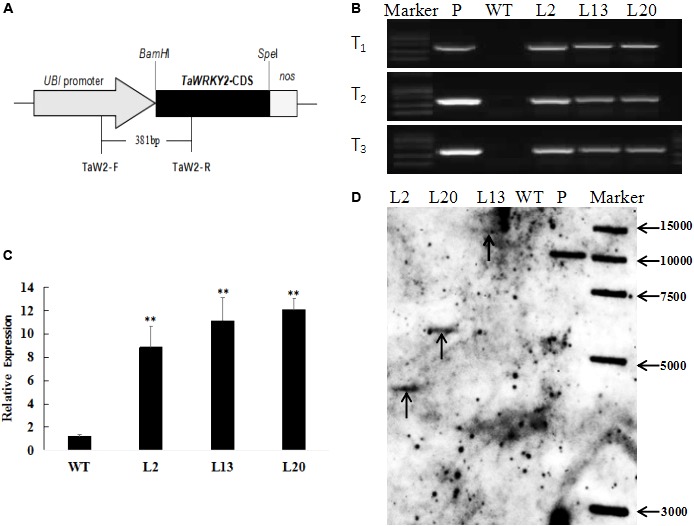
Molecular characterization of transgenic wheat. **(A)** Schematic diagram of the transformation vector pMWB122-*TaWRKY2*. **(B)** PCR analysis of the three transgenic lines from T_1_ to T_3_. **(C)** Relative expression of *TaWRKY2* in transgenic wheat. **(D)** Southern blot analysis of *Hind*III digested genomic DNAs from untransformed and T_3_ transgenic plants. WT, wild type; P, plasmid. Asterisks represent statistically significant difference compared WT (Student’s *t*-test, ^∗^*P* < 0.05, ^∗∗^*P* < 0.01).

To better understand the function of *TaWRKY2* in wheat, we generated transgenic wheat lines expressing *TaWRKY2* driven by an Ubi promoter. To identify the positive transgenic wheat lines, exogenous *TaWRKY2* was detected in the T_1_, T_2_, and T_3_ generations and confirmed by PCR using specific primers to amplify a 381-bp DNA segment spanning *UBI* promoter and *TaWRKY2* (**Figure 2A**). We isolated three independent transgenic lines (L2, L13, and L20) in which the exogenous *TaWRKY2* was authenticated from the T_1_ to T_3_ generations, whereas no amplification was detected in WT plants (**Figure 2B**). qRT-PCR analysis confirmed that the relative expression levels of *TaWRKY2* in the three transgenic lines mentioned above were significantly higher than the levels in WT plants (**Figure 2C**). As shown in **Figure 2D**, Southern blot analyses confirmed that the transformed *TaWRKY2* gene was indeed integrated into the genomes of lines L2, L13, and L20, with one copy present in each transgenic line (the unclear result for L13 in **Figure 2D**, supplemented with the blot shown in **Supplementary Figure S1**). These results confirmed that *TaWRKY2* was integrated into the wheat genome and expressed.

### *TaWRKY2* Enhances the Drought Tolerance of Transgenic Wheat at the Seedling Stage

**FIGURE 3:**
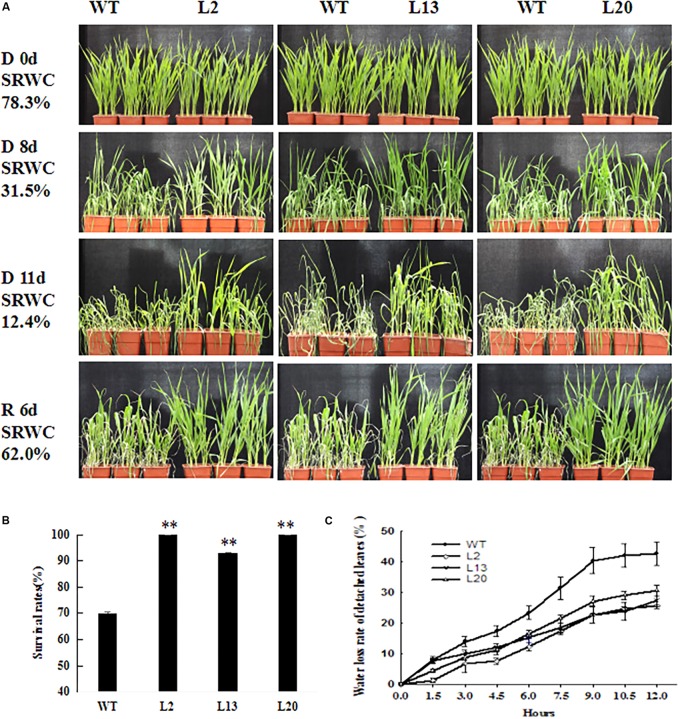
**(A)** Drought-resistant phenotypes. **(B)** Survival rates of WT and T_3_ *TaWRKY2* overexpressing lines. **(C)** Water loss rate of detached leaves. D 0d, drought for 0 days; D 8d, drought for 8 days; D 11d, drought for 11 days; R 6d, re-watering for 6 days; WT, wild type. Asterisks represent statistically significant difference compared WT (Student’s *t*-test, ^∗^*P* < 0.05, ^∗∗^*P* < 0.01).

To investigate the contribution of *TaWRKY2* to drought tolerance, 2-week-old seedlings of three independent T_3_ transgenic wheat lines (L2, L13, and L20) and WT wheat were subjected to water withholding (the LRWCs at each time point are shown in **Supplementary Table S3**). Before the drought treatment (drought treatment day 0, SRWC: 78.3%), there was no discernible difference between the WT and transgenic lines (**Figure 3A**). Eight days after drought treatment (SRWC: 31.5%), most leaves of all the transgenic lines remained upright, whereas those in WT plants started wilting (**Figure 3A**). After 11 days (SRWC: 12.4%), the transgenic wheat showed less wilting compared with WT (**Figure 3A**). After re-watering for 6 days (SRWC: 62.0%), almost all of the plants from the three transgenic lines recovered, with survival rates of 93–100%. In contrast, the WT wheat had a significantly low survival rate of 70% (**Figure 3B**). The water loss rate of detached leaves from the three transgenic wheat lines was significantly lower than that of the WT leaves at nine time points over a 12-h time course (**Figure 3C**). These results show that *TaWRKY2* significantly improved drought tolerance in the transgenic wheat seedlings.

### Drought Resistance Indexes in the Transgenic Wheat

**FIGURE 4:**
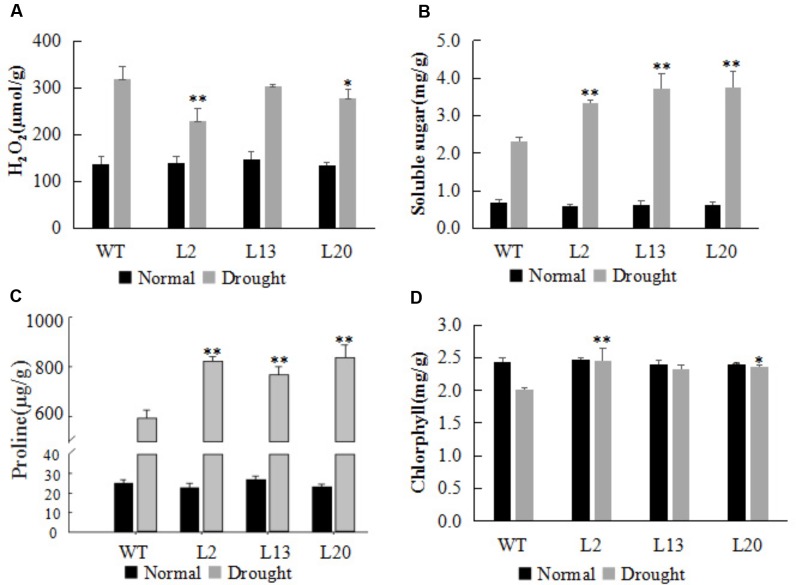
Changes in resistance indices in *TaWRKY2* overexpressing transgenic wheat **(A)** H_2_O_2_ content. **(B)** Soluble sugar content. **(C)** Proline content. **(D)** Chlorophyll content. Asterisks represent statistically significant difference compared WT (Student’s *t*-test, ^∗^*P* < 0.05, ^∗∗^*P* < 0.01).

The levels of osmolytes (free proline and soluble sugar), the accumulation of H_2_O_2_, and the chlorophyll content in leaves, have been widely used as important indicators of drought resistance (Szabados and Savoure, 2010). For all four parameters, the transgenic lines did not differ significantly from WT under normal growth condition (**Figure 4**). However, after drought treatment for 5 days, H_2_O_2_ accumulation in the leaves of transgenic plants was lower compared with WT (**Figure 4A**). The soluble sugar and free proline content were significantly enhanced in the transgenic wheat lines compared with WT, suggesting that *TaWRKY2* overexpression can help improve the accumulation of soluble sugar and proline in leaves under drought stress (**Figures 4B,C**). The chlorophyll content in the transgenic wheat lines was significantly higher than that in WT plants (**Figure 4D**). Thus, *TaWRKY2* overexpression led to physiological changes that may have contributed to the enhanced drought tolerance in the transgenic wheat lines.

### *TaWRKY2* Effects on Wheat Heading Under Drought Stress

**FIGURE 5:**
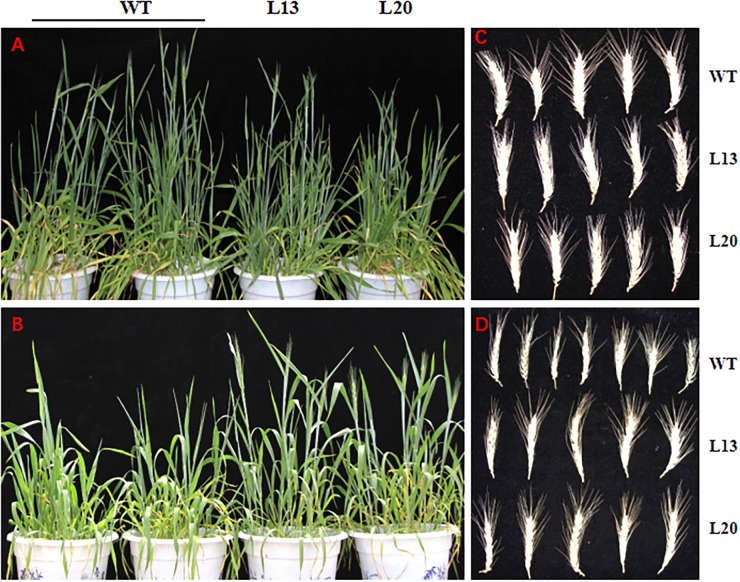
**(A)** Normal growth condition. **(B)** Drought stress for 20–25 days. **(C)** Wheat spikes under normal condition. **(D)** Wheat spikes under drought stress.

To test drought tolerance of transgenic wheat during heading stage, we applied drought treatment approximately 20–25 days prior to the wheat heading stage. The heading date of the transgenic wheat lines (L13 and L20) and WT plants were synchronous, and no significant differences were observed under normal conditions (**Figure 5A**). Although all of the plants had germinated at the same time, the transgenic plants progressed to the heading stage after drought treatment for 20–25 days, while WT growth was inhibited before heading (**Figure 5B**). After re-watering, the inhibitory effect of drought stress on the WT plants was removed. These results indicate that *TaWRKY2* overexpression could promote wheat heading under drought stress, which is beneficial in terms of life cycle completion under drought stress.

### *TaWRKY2* Transgenic Plants Had Increased Yield Under Drought Stress

Grain yield is an integrated parameter, and yield improvement is the ultimate goal of genetic engineering or conventional crossbreeding effects. After harvesting under both normal and drought conditions, some yield-related agronomic traits of *TaWRKY2* transgenic lines (L13 and L20) and WT were quantified. Under normal conditions, no significant differences in agronomic traits were found between the transgenic lines and WT plants, except for aboveground biomass between WT and L20 plants (**Table 1** and **Figure 5C**). Under drought stress, the aboveground biomass of transgenic line L13 increased slightly but not significantly compared with that of WT plants. However, the aboveground biomass of L20 was significantly greater than that of WT. Both transgenic lines had a longer panicle length (**Figure 5D**), more kernels per spike, and greater yield per plant compared WT. There was no significant difference in plant height between transgenic and WT plants under normal and drought stress conditions, respectively.

**Table 1:** Analysis of yield traits under normal and drought stress conditions.

Treatment	Lines	Aboveground biomass (g)	Plant height (cm)	Panicle length (cm)	Kernels per spike	Yield per plant (g)
Normal	WT	2.49 ± 0.13	51.13 ± 2.37	8.2 ± 0.37	25.33 ± 2.09	0.72 ± 0.05
	L13	3.0 ± 0.24	47.81 ± 2.03	8.01 ± 0.41	24.20 ± 2.67	0.78 ± 0.10
	L20	3.2 ± 0.32*	54.03 ± 1.85	8.02 ± 0.37	25.67 ± 3.69	0.73 ± 0.06
Drought	WT	1.54 ± 0.17	45.72 ± 1.33	6.69 ± 0.38	10.0 ± 1.92	0.31 ± 0.18
	L13	1.71 ± 0.19	44.96 ± 0.85	7.83 ± 0.29**	16.15 ± 2.2*	0.39 ± 0.17*
	L20	1.88 ± 0.19*	47.2 ± 0.28	8.62 ± 0.57**	16.11 ± 2.60*	0.48 ± 0.21*


*Values are indicated by mean ± SD (*n* = 10). Mean values of the transgenic lines are significantly different as compared with those in the WT lines (^∗^*P* < 0.05 and ^∗∗^*P* < 0.01).*

### *TaWRKY2* Activates the Expression of Drought Stress-Responsive Genes

**FIGURE 6:**
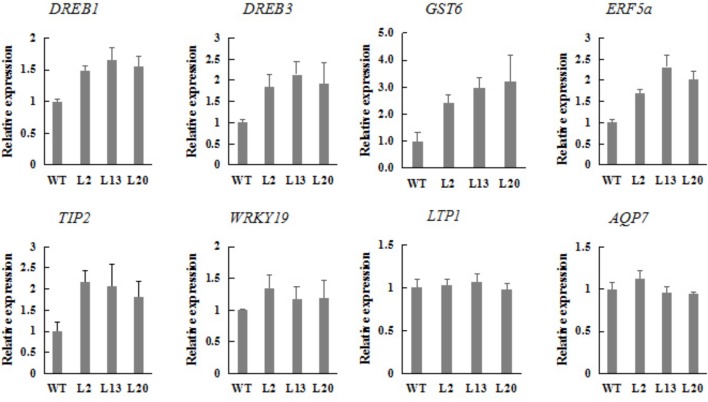
Expression of drought stress-related genes in *TaWRKY2*-overexpressing transgenic plants.

To investigate whether *TaWRKY2* overexpression affected the expression of stress-responsive genes, the expression of eight previously reported drought-responsive genes were examined in transgenic plants and WT wheat grown under normal condition using qRT-PCR (**Figure 6**).

Eight genes investigated encode two DREBs (*DREB1* and *DREB3*), a glutathione *S*-transferase (*GST6*), an ERF (*ERF5a*), a tonoplast intrinsic protein (*TIP2*), a WRKY (*WRKY19*), a lipid transfer protein (*LTP1*), an aquaporin (*AQP7*). Compared with WT, the *DREB1*, *DREB3*, *GST6*, *ERF5a*, *TaWRKY19*, and *TIP2* gene expressions were significantly enhanced in *TaWRKY2*-transgenic plants whereas *LTP1* and *AQP7* expressions were not significantly changed. These results indicated that *TaWRKY2* overexpression regulated the expression of the drought-related genes.

## Discussion

Technological advances have raised the possibility of improving drought tolerance in plants, potentially through the manipulation of TFs associated with stress responses. These TFs including ABRE-binding proteins, zinc finger proteins, NAM/ATAF/CUC, (NAC), V-myb avian myeloblastosis viral oncogene homolog (MYB), and APETALA2/ethylene responsive factor (AP2/ERF) can regulate a large number of downstream genes. Notably, a few of these TFs have been analyzed in studies using genetic engineering to improve tolerance to abiotic and biotic stresses (Agarwal et al., 2006). Previous research has firmly established that WRKY TFs play crucial roles in regulating plant abiotic stress responses (?; Jiang Y. et al., 2014; Kayum et al., 2014; Wang et al., 2014; Yan et al., 2014). Understanding the roles and mechanisms of wheat WRKYs in regulating stress responses is vital for understanding plant adaptations to environmental stress. Although a previous study showed that *TaWRKY2* expression was induced by multiple abiotic stresses and that *TaWRKY2* overexpression conferred tolerance to drought stress in transgenic *Arabidopsis* (Niu et al., 2012), no evidence was provided for the role of *WRKY2* in regulating drought response in wheat.

In the present study, the *TaWRKY2* promoter was analyzed for putative plant regulatory *cis*-elements using the Plant CARE database, and several regulatory *cis*-elements related to abiotic stress responses were identified. Histochemical GUS analysis demonstrated that the activity of the *TaWRKY2* promoter could be up-regulated by drought, salt, heat, and ABA but not cold, which is consistent with the previous finding that *TaWRKY2* expression was not induced by cold (Niu et al., 2012).

To explore the role of *TaWRKY2* in drought stress adaptations in wheat, we generated *TaWRKY2*-overexpressing wheat lines via transformation. The results of molecular characterization (PCR, qRT-PCR, and Southern blot) indicated that exogenous *TaWRKY2* was successfully transformed into acceptor wheat and that high *TaWRKY2* expression levels were stably inherited in the three transgenic lines from the T_1_ to the T_3_ generations. The transgenic wheat lines may therefore be used for further functional validating of *TaWRKY2* in wheat in future work.

Our drought tolerance assays showed that *TaWRKY2* overexpression significantly enhanced drought tolerance at the seedling stage in transgenic wheat, as evidenced by the higher survival rate and lower water loss rate of detached leaves compared with WT plants. Meanwhile, changes in physiological parameters, including H_2_O_2_ content, soluble sugars, proline content, and chlorophyll content after stress treatment, were better protected from oxidative and osmotic damages. These results suggest that *TaWRKY2* may serve as an important positive regulator for adaptations of wheat to drought stress at the seedling stage. During a prolonged exposure to drought stress before the heading stage, the growth of WT plants was inhibited, whereas the *TaWRKY2*-overexpressing lines were able to progress to the heading stage. Growth recovery was observed in WT plants only after re-watering. These results show that *TaWRKY2* overexpression can promote wheat heading to facilitate life cycle completion under drought stress.

Crop production is greatly impacted by complex environmental factors. Some studies have shown that overexpression of stress-related TFs may increase crop yield (Oh et al., 2009; Yu et al., 2013; Zhao et al., 2014; Shavrukov et al., 2016). However, little is presently known about the effects of WRKY TFs on wheat yield. Notably, the present findings demonstrate that *TaWRKY2* overexpression in wheat increased aboveground biomass and grain yield under drought stress. The transgenic wheat also had longer panicle length and more kernels per spike than WT plants under drought stress, indicating that these agronomic traits contributed to the increased yield.

The expression levels of several previously reported drought-responsive genes such as *DREBs*, *GST6*, *ERF5a*, *TIP2*, and *WRKY19* were up-regulated in the *TaWRKY2*-transgenic plants. The *DREB3* overexpression in transgenic wheat has been found to increase drought stress tolerance (Shavrukov et al., 2016). GST is ROS-Scavenging enzyme and is known to be involved in redox homeostasis of cells under various stress conditions (Huang et al., 2013). *ERF5a* and *TIP2* are involved in plant response to drought and salt stress (Wang et al., 2011; Eini et al., 2013). *WRKY19* positively contributes to plant tolerance to drought, salt, and cold stresses in transgenic *Arabidopsis* (Niu et al., 2012). These findings indicated that *TaWRKY2* contributes to drought stress tolerance in wheat possibly through the activation of a range of drought-related genes.

Overall, *TaWRKY2* promoted drought stress tolerance and increased the yield of wheat. This study provides a foundation for investigating wheat responses to drought stress and identifies a candidate target gene, *TaWRKY2*, for improving drought stress tolerance in wheat and other cereals.

## Author Contributions

HG contributed to experimental implementation, data analysis, and manuscript preparation. YW and PX contributed to experimental implementation. ZZ conceived and designed the study.

## Conflict of Interest Statement

The authors declare that the research was conducted in the absence of any commercial or financial relationships that could be construed as a potential conflict of interest.

## Footnotes

1 http://bioinformatics.psb.ugent.be/webtools/plantcare/html/

## Supplementary Material

FIGURE S1 Southern blot analysis of *Hind*III digested genomic DNAs from WT and T3 transgenic plants.

TABLE S1 Primer sequences used for PCR, qRT-PCR, and probes in this study.

TABLE S2 Distribution and function of *cis*-acting regulatory elements in *TaWRKY2* promoter.

TABLE S3 Leaf relative water content in WT and transgenic lines.
