# Supplementary figures and images for: Overexpression of a WRKY Transcription Factor TaWRKY2 Enhances Drought Stress Tolerance in Transgenic Wheat
Source: Front Plant Sci. 2018 Aug 7;9:997. doi: 10.3389/fpls.2018.00997 (PMC6090177; doi:10.3389/fpls.2018.00997)

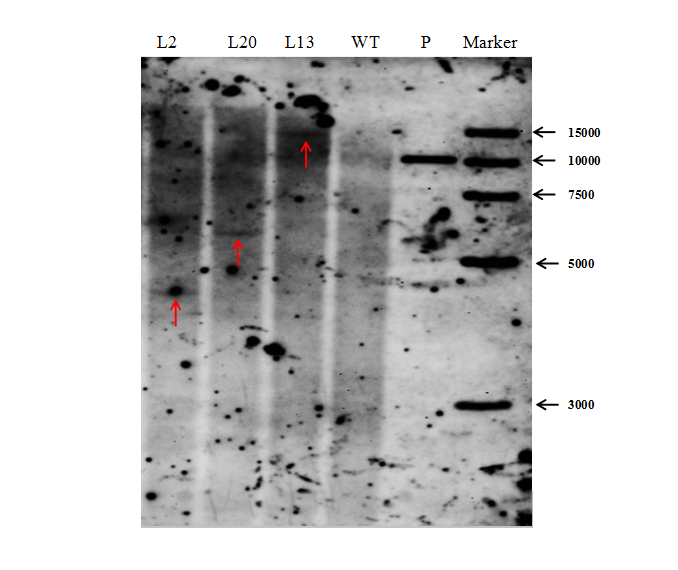

Supplement: FIGURE S1 — Southern blot analysis of HindIII digested genomic DNAs from WT and T3 transgenic plants. [file Image_1.PNG]
