# Supplementary material for: Overexpression of a WRKY Transcription Factor TaWRKY2 Enhances Drought Stress Tolerance in Transgenic Wheat
Source: Front Plant Sci. 2018 Aug 7;9:997. doi: 10.3389/fpls.2018.00997 (PMC6090177; doi:10.3389/fpls.2018.00997)
Supplement: TABLE S1 — Primer sequences used for PCR, qRT-PCR, and probes in this study. [file Table_1.doc]

**Table S1 | Primer sequences used for PCR, qRT-PCR and probes in this study**

| Name | Primer sequence ( 5’- 3’) |
| --- | --- |
| P_W2_-F/P_W2_-R | CAGACGGGATAAATAGGAAGTT/CATGGCGATCGACGCGACG |
| TaW2-F/TaW2-R | GCCTTCATACGCTATTTATTTGC/CCAGGATATGGGAGTAGTTGA |
| Ta2-F/Ta2-R | ATGTCCTCCTCCACGGGGAG/CTAGCAGAGGAGCGACT |
| QT-Ta2-F/QT-Ta2-R | GGCGCTGCCGACGTCATCTT/AGCAGAGGAGCGACTCGACGA |
| Taactin-F/Taactin-R | GGATCTGTATGGTAATGTTGTTCT/CACTGTATTTCCTTTCAGGTGGTG |
| UbiF/UbiR | TGCAGCGTGACCCGGTCGTGC/CAGCGTGACCCGGTCGTGC |
| *WRKY19*-F/*WRKY19*-R | AGGGAAGCATACGCATGACGTGC/GGCGAGATCGTTCAGAATGGCTGT  5’-GGCGAGATCGTTCAGAATGGCTGT-3’ |
| *DREB3* | CTCGATTCGCTTGCTCCTCAG/TCCTGATGACAAGCTGTAGTGTGC |
| *TIP2* | CCCCTACACACGGGCTTTC/TTGGTGAAGTCGCCGCTG |
| *GST6* | CATTTTGCTGGCAGTTTA/TGTTCGGTTGCTTGATGTAGT |
| *AQ7* | GGCCGGACTGAAGTGTAGAT/ACAGGACAAAGGTGTGGGAT |
| LTP1 | GCCCTCCACCACAACTACAG/CCGGCCAATCTCCTAACTCC |
| ERF5a | CTACACATCCGTCTTCGTTCC/CATCGCACCATTCAGCAGAA |
| DREB1 | AGACCGAGGCGAGAGGAGAT/GCAACCGAATCAGGACCAGTG |
