# Supplementary material for: Overexpression of a WRKY Transcription Factor TaWRKY2 Enhances Drought Stress Tolerance in Transgenic Wheat
Source: Front Plant Sci. 2018 Aug 7;9:997. doi: 10.3389/fpls.2018.00997 (PMC6090177; doi:10.3389/fpls.2018.00997)
Supplement: TABLE S2 — Distribution and function of cis-acting regulatory elements in TaWRKY2 promoter. [file Table_2.doc]

**Table S2 | Distribution and function of *cis*-acting regulatory elements in *TaWRKY2* promoter**

| **元件名称**  **Name** | **序列**  **Sequence** | **功能**  **Function** | **位置**  **Location** |
| --- | --- | --- | --- |
| A-box | CCGTCC | 厌氧诱导元件 | -626~-621, |
| ABRE | CACGTG | ABA应答元件 | -214~-209(-) |
| 3-AF1binding site | AAGAGATATTT | 光应答元件 | -815~- 806(-) |
| ARE | TGGTTT | 分生组织作用元件 | -1359~-1354,-162~-157(-) |
| AT-rich element | AT ATAAATCAA | ATBP-1结合位点 | -778~-768 |
| ATC-motif | TGCTATCCA | 光应答元件一部分 | -1197~-1189 |
| BOX-W1 | TTGACC | 真菌诱导子应答元件 | -1980~-1985 |
| CATT-motif | GCATTC | 光应答元件的一部分 | -1899~-1984,-602~-597(-) |
| CAT-BOX | GCCACT | 分生组织表达元件 | -1043~-1038,-1918~-1914 |
| CCAAT-box | CAACGG | MYBhv1结合位点 | -641~-636 |
| CGTCA-box | CGTCA | MeJa 响应元件 | -235~-231 |
| EIRE | TTCGACC | 激发子应答元件 | -1839~-1833 |
| GAG- motif | GGAGATG | 光应答元件一部分 | -1766~-1760,-1627~-1618, -1482~-1476 |
| GARE-motif | AAACAGA | gibberellin-responsive element | -88~-82(-) |
| G-box | CACGTT | 光应答顺式作用元件 | -255~-250,-1296~-1301(-)  -1191~-1185(-) |
| GCN4_motif | TGTGTCA | 胚乳表达作用元件 | -1188~-1182 |
| GTGGC-motif | GCCACACGA GG | 光应答元件 | -1595~-1585,-1353~-1343 |
| HSE | AAAAAATTTC | 热胁迫响应元件 | -957~-948(-) |
| I-BOX | CTCTTATGCT | 光应答元件 | -899~-890(-) |
| MNF1 | GTGCCC(A/T) | 光应答元件 | -947~-941 |
| MBS | TAACTG/CAGTTA | 相应干旱胁迫位点 | 1641-1636(-),-232~-227 |
| O2-site | GATGATGTGG/  GTTGACGTGA | 新陈代谢调控元件 | -1922~-1913,-238~-229(-) |
| P-box | CCTTTTG | gibberellin-responsive | -1657~-1651(-) |
| SP1 | CC(G/A)CCC/  GGGCGG | 光应答元件 | -192~-183,-327~-322(-) |
| TATCCAT/C-motif | TATCCAT | 未知 | -1194~-1188 |
| TCA-element | GAGAAGAATA | 水杨酸响应元件 | -1015~-1007(-),-263~-254(-) |
| TCCC-motif | TCTCCCT | 光应答元件 | -31~-25 |

“-” represnets negative strand.
