# Supplementary material for: Overexpression of a WRKY Transcription Factor TaWRKY2 Enhances Drought Stress Tolerance in Transgenic Wheat
Source: Front Plant Sci. 2018 Aug 7;9:997. doi: 10.3389/fpls.2018.00997 (PMC6090177; doi:10.3389/fpls.2018.00997)
Supplement: TABLE S3 — Leaf relative water content in WT and transgenic lines. [file Table_3.doc]

**Table S3 | Leaf relative water content in WT and transgenic lines**

| Lines | 0 day(%) | 8 days(%) | 11days(%) | R-6days(%) |
| --- | --- | --- | --- | --- |
| WT | 84.7±0.78 | 49.1±2.69 | 25.7±3.47 | 80.2±1.16 |
| L2 | 87.2±2.96 | 75.7±1.04 | 62.8±3.91 | 79.1±2.37 |
| L13 | 85.0±7.03 | 70.5±4.46 | 64.1±6.64 | 83.4±0.96 |
| L20 | 89.1±1.95 | 73.7±5.56 | 60.7±1.93 | 82.8±6.95 |

**(1)Leaf relative water content**

LRWC(%)=(FW-DW)/(SFW-DW)×100

**LWC:** Leaf relative water content;**FW:** Fresh weight;**SFW:** Saturated fresh weight;**DW:** Dry weight

**(2)Soil relative water content**

SRWC(%)=SWC/SSWC×100

**SRWC:** Soil relative water content;**SWC:** Soil water content;**SSWC:** Soil saturated water content
